# Parkinson’s Disease Caregiving, Level of Care Burden, Caregiving-Related Strain, and Caregiver Health

**DOI:** 10.3390/healthcare13131520

**Published:** 2025-06-26

**Authors:** Julie S. Olson, Reema Persad-Clem, George C. Kueppers, Fawn A. Cothran, Margaret L. Longacre

**Affiliations:** 1 College of Health Sciences, Arcadia University, Glenside, PA 19038, USApersadclemr@arcadia.edu (R.P.-C.); 2 National Alliance for Caregiving, Washington, DC 20036, USA; 3 Independent Researcher, Greenbelt, MD 20768, USA

**Keywords:** Parkinson’s disease caregiving, care burden, caregiving-related strain, caregiver health

## Abstract

**Background:** Caregiving can be a challenging experience, particularly for caregivers of people with Parkinson’s disease, given the array of motor and neuropsychiatric symptoms. Elevated care tasks and demands related to these symptoms may result in greater care burden, heightened caregiving-related strain, and, in turn, poorer health for Parkinson’s disease (PD) caregivers compared to non-PD caregivers. Guided by the Stress Process Model, the purpose of this study was to explore the pathways connecting PD caregiving and caregiver health, with attention to the role of care burden and caregiving-related strain. **Methods:** We applied path analysis in a structural equation modeling framework to data from 3116 PD and non-PD caregivers participating in the National Alliance for Caregiving and AARP’s *Caregiving in the U.S.* 2015 and 2020 surveys. We estimated pathways between PD caregiving, care burden, caregiving-related strain (i.e., emotional, physical, and financial), and caregiver self-reported health simultaneously, then decomposed these pathways into total, indirect, and direct effects. **Results:** Findings show PD caregiving is indirectly linked to poorer health among caregivers through increased care burden and heightened caregiving-related strain, with additional path analysis models pointing to physical strain as an important component of caregiving-related strain in mediating the associations between PD caregiving and overall health. **Conclusions:** Our findings suggest a need to be especially attentive to the accumulation of care burden and caregiving-related strain—particularly physical strain—among PD caregivers, given the potential consequences for caregiver health. Solutions are needed, such as caregiver screening and caregiver-specific care plans, to better support reductions in burden and strain among PD caregivers, thereby promoting their overall health.

## 1. Introduction

According to research by the National Alliance for Caregiving and AARP, approximately 53 million Americans—21.3% of the United States (U.S.) population—were providing care to a friend or relative in 2020 [1]. Of caregivers in the 2015 and 2020 samples of the NAC and AARP *Caregiving in the U.S.* datasets, an estimated 2% were caring for a friend or family member who had been diagnosed with Parkinson’s disease (PD) [2]. Affecting over a million people, PD is the second most common neurodegenerative disease in the U.S. (after Alzheimer’s disease). and its prevalence is expected to increase by 2040 [3,4]. As PD prevalence continues to rise, attention must be given to the potential impacts of PD caregiving on caregiver health outcomes.

Caregiving, in general, can be demanding, and as such, caregivers often report poorer physical and psychosocial health than non-caregivers [5,6,7,8,9]. PD, in particular, involves deficits in motor function, including resting tremors and rigidity, as well as neuropsychiatric symptoms such as depression, anxiety, and psychosis, especially in later stages of the disease [3,10,11,12,13]. The unique characteristics of PD, the intensive care that PD requires, and the highly variable presentation of symptoms may, therefore, place PD caregivers at even greater risk of poor health outcomes [14,15]. To better understand this risk and identify the pathways through which PD caregiving influences overall health, the Stress Process Model is one guide [16,17]. This framework theorizes that experiences of stress related to caregiving impact environmental demands for caregivers; then, environmental demands contribute to perceived stress; and finally, perceived stress places caregivers at risk for poorer health outcomes.

Guided by the Stress Process Model, assessing connections between PD caregiving and caregiver health, while considering pathways through environmental demands (i.e., objective care burden) and perceived stress (i.e., caregiving-related strain), offers an approach to examine PD caregiver health risk and pathways. Objective care burden refers to the measurable time spent providing care and the tangible tasks for which caregivers are responsible [18,19,20]. Care tasks that require considerable time can include, but are not limited to, dressing or bathing, doing the laundry or grocery shopping, assisting with mobility issues, and managing medication. Experiences of burden thus vary by dependency of care recipients and intensity of care being provided [21,22]. Caregiving-related strain refers to the physical, emotional, and financial stress felt by the caregiver as a direct result of their caregiving duties [23]. Caregiver burden and caregiving-related strain are inter-connected and have been shown to negatively influence caregivers’ health [24,25]. As PD progresses, reliance on caregivers increases due to greater patient dependence, loss of motor control, decline in cognitive ability, and behavioral changes [26]. PD progression is associated both with greater burden and heightened caregiving-related strain [27,28] and may increase the salience of the stress process pathways for PD caregivers compared to non-PD caregivers.

Understanding the implications of care burden and caregiving-related strain among PD caregivers is central to the development of interventions, programs, or policies to improve the health and well-being of caregivers. Emerging literature documents experiences of PD caregiving and elucidates the predictors and contributors to caregiver burden, including patient (e.g., neuropsychiatric symptoms), caregiver (e.g., relationship to the care recipient), and relational or sociocultural factors (e.g., relationship to the care recipient, poor access to information, limited clinical support) [18,29,30,31,32]. Previous work also suggests that PD caregiver burden and strain are compounded by financial pressures, shifts in social connectivity, changes in the caregiver-recipient relationship, and worsened health due to increased need for physical support and diminished self-care [18,33].

Although attention to the demands of PD caregiving has increased, more research is needed to explore the ways that these caregiving experiences might jeopardize caregiver health. The purpose of this study was to explore associations between the presence of PD, caregiver burden, caregiving-related strain, and overall caregiver health. We hypothesized that, due to demands related to motor and non-motor function, PD caregivers would report elevated care demands compared to non-PD caregivers, and that the heightened burden of these care demands would result in higher perceived caregiving-related strain for PD caregivers; in turn, caregiving-related strain would negatively impact caregivers’ perception of their overall health. In other words, by applying the Stress Process Model to data from PD and non-PD caregivers, we expected to find indirect pathways linking PD caregiving and overall health through objective care burden and caregiving-related strain.

## 2. Methods

### 2.1. Data Source and Sample

The National Alliance for Caregiving’s *Caregiving in the U.S.* is a nationally representative study designed to describe the experiences, characteristics, and needs of adult American caregivers. Using a national, probability-based, online tool, KnowledgePanel^®^, quantitative online survey data were collected for *Caregiving in the U.S.* 2015 and *Caregiving in the U.S.* 2020. Respondents were aged 18 years or older and reported that they provided unpaid care currently (at the time of the survey) or in the last 12 months. All caregivers also had to be providing help with at least one Activity of Daily Living (ADL), Instrumental Activity of Daily Living (IADL), or medical/nursing task.

Our analytical sample was drawn from a combined sample of 3116 caregivers who participated in the 2015 or 2020 study and reported caring for an adult care recipient. A core set of questions about caregiving experiences was designed to match between survey years. The maintained methodology across survey years and consistent questions allowed for responses to be combined. All caregivers in our sample had sampling weights, which were calculated using a population-based weighting process by age, sex, and race/ethnicity.

### 2.2. Measurement

#### 2.2.1. Experience of Parkinson’s Disease Caregiving

In 2015 and 2020, all caregivers were asked to report the primary reason for which the care recipient required care. In 2015, an additional survey item asked caregivers whether the care recipient had PD. Thus, we categorized 2015 caregivers as PD caregivers based on both survey items (*n* = 27 for primary reason; *n* = 22 for another primary reason but PD diagnosis). The additional question regarding PD was not asked in 2020, so for that survey year, we only classified caregivers as PD caregivers if they noted PD as the primary reason for which the recipient required care (*n* = 23). Across the 2015 and 2020 surveys, 72 caregivers indicated PD as a reason for providing care, with 3044 respondents providing care for a reason other than PD.

#### 2.2.2. Caregiver Sociodemographic and Caregiving Characteristics

Participants provided information on age, gender identity, race and Hispanic ethnicity, marital status, geographic location, educational attainment, household income, and employment status. Participants also self-reported on time spent caregiving (occasionally, less than 6 months, 6 months to 1 year, 1 to 4 years, 5 to 9 years, or 10 or more years), whether they were currently providing care at the time of the survey, and whether the care recipient lives with the caregiver.

#### 2.2.3. Care Burden

Care burden was measured using a *Level of Care Index*, which is a series of caregiver responses on time and tasks representing objective caregiver burden. The number of hours of care and number of ADLs (e.g., bathing, toileting) and IADLs (e.g., paying bills, transportation) are combined into a five-point burden score, with level 1 representing the lowest caregiver burden and level 5 indicating the highest. Importantly, operationalizing care burden using the *Level of Care Index* allowed for measurement of objective care burden.

#### 2.2.4. Caregiving-Related Strain

Caregivers responded to questions on caregiving-related emotional, physical, and financial strain (i.e., how straining/stressful caregiving was for them on a scale of 1: *Not at all* to 5: *Very much)*. Here, we not only used each of the three indicators of strain individually, but we also calculated a total score for overall strain by summing these questions. For total caregiving-related strain, higher scores indicated greater strain. Caregiving-related strain scores have been shown to be psychometrically sound [34] and were validated against other subjective assessments of caregiver experience, including, for example, the Zarit Burden Interview [35].

#### 2.2.5. Caregiver Self-Rated Health

Caregiver health was captured using a survey-administered self-rated health measure. Specifically, all respondents were asked how to describe their own health status at the time of caregiving as poor, fair, good, very good, or excellent. Caregiver self-reported health was then collapsed to create a dichotomous indicator where 1 = poor or fair health and 0 = good, very good, or excellent health. Dichotomizing caregiver health in this way allowed us to capture the respondents in our sample facing the highest risk for poor health outcomes.

### 2.3. Analysis

Descriptive statistics were calculated for the full sample and by whether the respondent was providing care to a person(s) with PD. We examined bivariate differences between PD caregivers and non-PD caregivers using *t*-tests and chi-square tests, and we report statistically significant differences (*p* < 0.05). We also report bivariate differences in sociodemographic and caregiving characteristics by caregiver health status (poor or fair health vs. good, very good, or excellent health).

**Figure 1:**
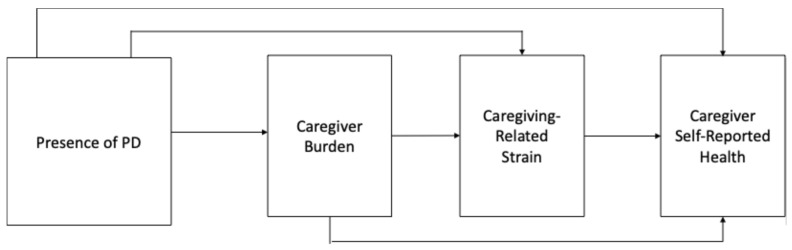
Conceptual model of presence of PD, caregiver burden, caregiving-related strain, and caregiver health.

The primary goal of this study was to understand the dynamic linkages between PD caregiving, care burden, total caregiving-related strain, and caregiver health (conceptual model in Figure 1). To address this goal, both direct and indirect pathways were tested between PD caregiving and poor or fair caregiver health through care burden and caregiving-related strain in a single structural equation model using path analysis. Thus, we estimated all pathways between PD caregiving, burden, strain, and health simultaneously, and decomposed the pathways’ total effects into direct and indirect effects. To further investigate these pathways and better understand how the components of total strain contribute to the associations between PD caregiving and health, three distinct models were estimated, each using one component of the total caregiving-related strain score (i.e., financial strain, emotional stress, and physical strain).

Regression and mediation models were estimated using structural equation modeling in the statistical software STATA (v. 18.5). Full information maximum likelihood (FIML) estimated exogenous variance for missingness such that all cases in the sample were retained in multivariable models, even if they had missing data on individual variables. Covariate effects were included in all structural equation models by controlling for the relationships between sociodemographic background and caregiving characteristics, and caregiver health. Sampling weights were applied in all descriptive and multivariable analyses. Standardized regression coefficients are reported for path analysis results.

## 3. Results

### 3.1. Caregiver Sociodemographic, Caregiving Characteristics, and Caregiver Health

Sociodemographic and caregiving characteristics are reported in Table 1 for the full sample of caregivers and according to PD or non-PD caregiving status. Most of the full sample of caregivers identified as female (59%) and non-Hispanic White (65%) and reported an average age over 50 (52.5 years). Most respondents were married (58%), and 14% resided in rural locations. Over one-third of respondents (35%) had at least a Bachelor’s degree, 40% reported household incomes of USD 75,000 or greater, and half were employed at the time of the survey. Most caregivers in the sample were currently providing care (60%), and 28% had been providing care to the care recipient for at least 5 years.

Compared with non-PD caregivers, PD caregivers were older (*M* = 60.5 years; *p* < 0.001), more likely to be women (71%; *p* < 0.05), and married (78%; *p* < 0.05). The proportion of caregivers currently providing care was significantly higher among PD caregivers (74%) compared with non-PD caregivers (60%). Additionally, more PD caregivers (55%) than non-PD caregivers (41%) were living with the care recipient, a statistically significant difference (*p* < 0.05).

When considering the full sample, 18% of caregivers rated their health as poor or fair. Among PD caregivers, 22% reported poor or fair health, and among non-PD caregivers, 18% reported poor or fair health. Compared to caregivers with good, very good, or excellent self-rated health, caregivers with poor or fair health were significantly less likely to be non-Hispanic White (61%), married (50%), and currently employed (36%) than caregivers with more advantaged health outcomes (66% White, 60% married, 53% employed). The caregivers in the full sample who rated their health as poor or fair also reported significantly lower levels of educational attainment and household income, on average, than caregivers with better health. Additionally, 53% of caregivers with poor or fair health reported living with the care recipient, compared with 38% of caregivers with good, very good, or excellent health (*p* < 0.001).

### 3.2. Care Burden, Caregiving-Related Strain, and Association with Caregiver Health

Table 2 provides descriptive statistics for care burden, caregiving-related strain, and self-reported caregiver health for the full sample and for PD and non-PD caregivers. Care burden was significantly higher among PD caregivers compared with non-PD caregivers (*p* < 0.001). Indeed, the highest level of care burden was reported by 15% of non-PD caregivers compared with 25% of PD caregivers. Total caregiving-related strain was also significantly higher among PD caregivers than non-PD caregivers (*p* < 0.001). Considering the three components of total strain, emotional stress and physical strain were significantly higher among PD caregivers than non-PD caregivers, but no significant difference was seen for financial strain.

**Figure 2:**
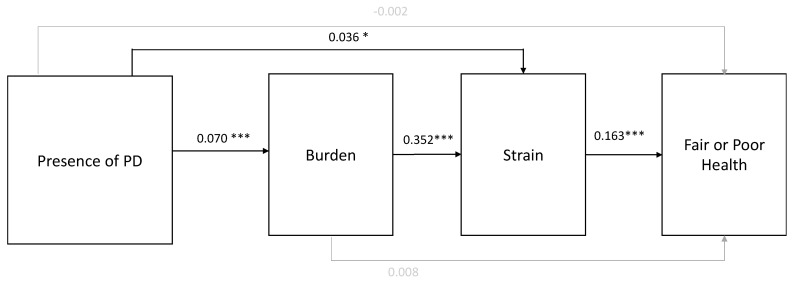
Results of structural equation modeling. Note: PD = Parkinson’s disease; standardized coefficients shown; all pathways shown with gray lines representing pathways that were not statistically significant; * *p* < 0.05, *** *p* < 0.001.

Path analysis models are shown in Figure 2. The direct pathway between PD caregiving and poor/fair health in the model was not statistically significant. A significant indirect pathway was shown between PD caregiving and health via burden and total strain (i.e., the summary score of emotional, physical, and financial strain). Specifically, PD caregivers reported greater care burden (β = 0.070; *p* < 0.001), which was linked to heightened perceived caregiving-related strain summary score directly (β = 0.036; *p* < 0.05) and through care burden (β = 0.352; *p* < 0.001). Greater care burden, in turn, was associated with the likelihood of reporting poor or fair self-rated health among caregivers (β = 0.163; *p* < 0.001). Thus, higher care burden among PD caregivers was associated with more caregiving-related strain, which then jeopardized caregiver self-perception of health. Similarly, PD caregiving was directly associated with greater perceived caregiving-related strain summary score, which then impacted perceptions of the caregivers’ health.

The total effects for these pathways were then broken down into direct and indirect effects; the results are reported in Table 3. These results provide further support for the findings described above and depicted in Figure 2. Indeed, there is strong evidence of indirect associations between PD caregiving and perceived health such that the increased care burden and caregiving-related strain reported by caregivers were associated with overall self-reported health. Importantly, the breakdown of total effects into direct and indirect effects also highlighted that PD caregiving was linked to strain both directly and indirectly through increased burden. In sum, these results document the downstream impacts of PD caregiving (compared to non-PD caregiving) on caregivers’ self-reported health through the heightened care burden and, subsequently, perceptions of caregiving-related strain. Experiences of greater caregiving-related strain, in particular, contribute most directly to the likelihood of poor or fair health.

### 3.3. Detailed Assessment of Caregivers’ Emotional, Physical, and Financial-Related Caregiving Strain

Given the importance of caregiving-related strain in the pathways documented above, further analysis explored the importance of caregiving-related financial strain, emotional stress, and physical strain (i.e., the three components of the total strain score) by running the path analysis separately for each of the three component scores to capture strain. Total, direct, and indirect effects for these pathways are shown in Table 4. In the model with financial strain, there is evidence of a significant indirect pathway from PD caregiving to caregiver health via burden and financial strain. No direct link was found between PD caregiving and financial strain; however, there was a pathway linking PD caregiving and poorer perceived health through heightened care burden and heightened financial strain, as shown in Table 4. Similarly, in the model with emotional stress as the indicator of strain, we found evidence only of an indirect pathway from PD caregiving to burden and then, subsequently, emotional stress and caregiver health. When strain was operationalized as physical strain in the path analysis, PD caregiving was linked to physical caregiving-related strain both directly and indirectly through increased burden.

In summary, each of the three caregiving-related strain components (i.e., physical, emotional, and financial) mediated the association between burden and caregiver health, and PD caregiving was indirectly linked to caregiver health through the cumulative pathway of burden and financial/emotional/physical strain. PD caregiving was also associated directly with strain only when physical strain was considered, emphasizing physical strain as an important experience of PD caregiving that is significantly associated with caregiver health.

## 4. Discussion

PD caregiving can be uniquely demanding—both physically and emotionally—and as such, puts caregivers at risk for poor health outcomes [17]. Guided by the Stress Process Model, we explored pathways between PD caregiving, care burden, caregiving-related strain, and caregiver health in a structural equation modeling framework to gain insights into the ways that PD caregiving might be linked to overall health. Consistent with our hypothesis and in line with theorized mechanisms, findings from the present study suggest that experiences of care burden and caregiving-related strain, which are particularly salient for PD caregivers, contribute to poorer health outcomes.

Importantly, our results did not support a direct effect between PD caregiving and overall self-rated health. Instead, our exploratory model revealed increased burden and heightened strain indirectly link PD caregivers to poorer health. PD caregiving was significantly associated with greater burden and, independently, more caregiving-related strain, compared with non-PD caregivers. In turn, burden was associated with strain, and strain was significantly associated with poorer self-reported health. These results, therefore, highlight the experiences of burden and strain as both critical to overall health and particularly salient for PD caregivers compared to non-PD caregivers. These findings are consistent with previous research that documents profound experiences of burden and strain among PD caregivers [18,31,36], and further extend this literature to better illustrate the means by which these experiences impact health. Overall, the documentation of these linkages points to the vital need for practical support and policy recommendations that might reduce caregiver burden and caregiving-related strain among PD caregivers.

Specifically, reducing care burden may be critical to lowering strain and improving health outcomes among PD caregivers. Programs, policies, and logistical support to decrease time spent and tasks performed by caregivers may serve as mechanisms through which caregiver burden can be reduced. Indeed, mechanisms to lower burden could be both through internal coping skills (e.g., resilience) and external networks (e.g., social support). Caregiver resilience, for example, is protective against burden and distress [37], enabling caregivers to maintain emotional stability and problem-solve effectively. Social support may also provide caregivers emotional validation, instrumental help, and opportunities for respite, thus lowering care burden [38]. These internal and external buffers often function synergistically, as social support enhances resilience by providing emotional and practical resources [39]. Thus, programs that combine the development of skill-building with social networks, such as REACH II [40], may be particularly effective in reducing burden and ultimately lowering caregiver risk for poor health outcomes [41].

Our findings also illustrate the importance of caregiving-related strain due to its role in mediating not only the association between PD caregiving and health but also the association between burden and health. Thus, to better understand this mechanism, we considered the three components of total strain individually (i.e., physical strain, emotional stress, and financial strain). As a result, our analysis pointed to physical strain as a particularly critical experience that impacts perceived stress. This finding echoes previous research highlighting the importance of the physical component of PD caregiving given the progressive nature of PD and the symptomatology related to loss of motor function [42]. In our sample, PD caregivers were significantly more likely than non-PD caregivers to report helping the care recipient with all activities of daily living (ADLs), including but not limited to getting in and out of beds and chairs, getting dressed, getting to and from the toilet, and bathing or showering. In addition to contributing to strain, these tasks can jeopardize health by putting caregivers at risk for injury [43]. PD caregivers may benefit from more accessible resources and support to assist them in care tasks and provide needed respite. Patient education for PD caregivers could also be better curated by disease stage to inform these caregivers about what to expect and how to prepare for anticipated changes as the disease progresses [29].

As PD caregivers navigate the physical and psychosocial demands of their role, we must also consider the multifaceted ways in which they view their own health and well-being. In our analyses, we used self-rated health to operationalize caregiver health, but other measures of health perception can give further insight into the ways caregiving might matter for caregivers’ health outcomes. For example, the subsample of our respondents who were surveyed in 2020 were asked if they found it difficult to take care of their own health. Significantly more PD caregivers (45%) than non-PD caregivers (23%) agreed or strongly agreed. Caregivers in *Caregiving in the U.S.* were also asked how caregiving impacted their health. A PD diagnosis necessitates long-term care [44]; thus, over the course of their caregiving journey, if caregivers either neglect their own health needs or note worsening health, their risk of poorer health may compound over time. Future research should, therefore, continue to evaluate markers of health perception among PD caregivers and seek to understand how these markers might be associated with both short- and long-term outcomes.

### 4.1. Recommendations

Based on these findings, we present two practice-level recommendations that have the potential to better assess and mitigate PD caregiving-related strain, thus identifying risk earlier and mitigating negative health outcomes for PD caregivers. First, caregivers should be screened in clinical care settings to assess care burden and caregiving-related strain. Strain due to the progressive worsening of symptoms in the care recipient can result in increased PD caregiver depressive symptoms, suicidality, and mortality [45]. The broad implementation of clinical screening tools [29] to evaluate strain and its components can, therefore, better identify and support PD caregivers who are at greatest risk. One example of a clinical screening tool that has been used in cancer care is the “Distress Thermometer” [46]. This brief screening tool requires about 3 min of time, and caregivers indicate their level of distress over the past week on a scale of 0–10, with zero indicating no distress and 10 indicating extreme distress. Based on initial findings, supplementary actions to better support caregiver well-being can be implemented, and the screening tool can be used during future visits to identify new changes or improvements over time. Instruments specifically designed for use with PD caregivers can further assess individual caregiver strain relevant to some of the demands of PD caregiving, including hypervigilance or safety, as well as more family-level measures (e.g., Parental Illness Impact) to reflect the wide network often necessary to support PD caregivers [47]. Research should continue to evaluate and adapt existing instruments, such as the National Alliance for Caregiving’s caregiving-related strain measure, in collaboration with PD caregivers to ensure generalizability and applicability of these tools across a range of sociodemographic groups [48].

Second, to better support the health of PD caregivers, we recommend implementing person-centered care (PCC) approaches [49]. PD caregivers could benefit from caregiver-specific care plans. Clinicians can assess PD caregiver quality of life (e.g., BELA-A-k) [50] and discuss resources centered on caregiver well-being at relevant stages of disease progression. For example, earlier-stage resources can emphasize the importance of self-care [18], caregiver-specific cognitive behavioral therapy, and connecting with other caregivers (e.g., support groups), while later-stage resources can involve guidance oriented towards accessing local and federal support and palliative care [29]. In a PCC model, moreover, caregivers build relationships with the care team, ask questions, and receive support, ultimately bolstering their knowledge of and preparedness for caregiving tasks [15,51]. Ensuring caregivers are prepared to care for their loved ones not only benefits the patient but also promotes caregiver quality of life [52,53,54]. Taken together, engaging with patients and informal caregivers in centralized PCC models that support caregivers through personalized training, resources, and support can translate to improved outcomes for both the patient and the caregiver. Further work considering burden, strain, and health among PD caregivers could also benefit from a dyadic study design.

### 4.2. Limitations

The analytical sample in this study was pulled from combined data of the 2015 and 2020 *Caregiving in the U.S.* surveys. In both survey years, respondents were asked to select the primary reason for which they were providing care. In 2015 only, an additional question asked caregivers if the care recipient had been diagnosed with PD, even if PD was not selected as the primary reason for providing care. Given that this additional PD question was not asked in 2020, caregivers in that survey year were only classified as PD caregivers if they reported PD as the primary reason for providing care. Our results may, therefore, be an underrepresentation of PD caregiving. PD caregivers, furthermore, represented only 2% of the total sample, and this imbalance reduces statistical power, increasing the likelihood that true differences may go undetected. Though sampling weights were applied in all analyses, the generalizability of our findings may still be limited due to sample size. Continued efforts to understand caregiving experiences should consider oversampling PD caregivers. The survey was cross-sectional and relied on self-reported data. Additionally, analytical models did not specify the relationship between the caregiver and the care recipient. Experiences of burden and psychosocial distress in caregiving vary by spousal versus adult-child caregivers, for example [55]. Future research should, therefore, explore the moderation of the pathways we document by the relationship of the caregiver to the person with PD. Finally, caregiving research has broadly suggested that the COVID-19 pandemic exacerbated caregiver experiences of burden and increased the intensity of care provided by informal caregivers [56]. Given that the analytical sample in this study was surveyed prior to the pandemic, additional work is needed to understand the ways in which experiences of burden, strain, and caregiver health have continued to evolve since 2020.

## 5. Conclusions

By applying a Stress Process framework to data on PD caregiving and caregiver health, this research documents indirect pathways linking PD caregivers to poorer health through heightened care burden and increased caregiving-related strain. Burden and strain—particularly physical strain—are common among PD caregivers, and thus these findings highlight the need to provide PD caregivers with resources and supports to reduce their experiences of care burden and caregiving-related strain and, ultimately, mitigate the health impacts of caregiving.

## Figures and Tables

**Table 1:** Sociodemographic and caregiving characteristics among Parkinson’s disease (PD) and non-PD Caregivers.

	Full Sample	Non-PD Caregivers	PD Caregivers	Between Group Differences
	*n* = 3116	*n* = 3044	*n* = 72	
	%/*M* (*SD*)	%/*M* (*SD*)	%/*M* (*SD*)	
**Gender identity**				
Male	41%	41%	29%	*
Female	59%	59%	71%	
**Age**	52.5 (18.5)	52.3 (18.5)	60.5 (17.0)	***
**Race/ethnicity**				
Non-Hispanic White	65%	65%	67%	
Non-Hispanic Black	13%	13%	11%	
Asian/Pacific Islander, non-Hispanic	5%	5%	5%	
Other	3%	3%	2%	
Hispanic	15%	15%	15%	
**Marital status**				*
Married	58%	58%	78%	
Living with partner	7%	7%	6%	
Widowed	6%	6%	2%	
Separated	2%	2%	0%	
Divorced	8%	8%	7%	
Single, never married	18%	19%	7%	
**Rural**	14%	14%	8%	
**Educational attainment**				
Less than high school	7%	7%	13%	
High school grad/GED	25%	26%	22%	
Some college	23%	23%	16%	
Technical school, Associate’s degree	9%	10%	7%	
Bachelor’s degree	20%	21%	18%	
Graduate or Professional degree	15%	15%	25%	
**Household income**				
Under USD 15 k	9%	9%	6%	
USD 15 to USD 29 k	15%	14%	24%	
USD 30 to USD 49 k	18%	18%	16%	
USD 50 to USD 74 k	18%	18%	11%	
USD 75 to USD 99 k	13%	13%	21%	
USD 100 k+	27%	27%	22%	
**Currently employed**	50%	50%	44%	
**Currently caregiving**	61%	60%	74%	*
**Time providing care**				
Less than 6 months	26%	27%	13%	
6 months to 1 year	19%	19%	23%	
1 to 4 years	27%	27%	29%	
5 to 9 years	14%	14%	18%	
10+ years	14%	14%	17%	
**Lives with care recipient**	41%	41%	55%	*

Note: *M* = mean, *SD* = standard deviation; * *p* < 0.05; *** *p* < 0.001.

**Table 2:** Caregiver burden, caregiving-related strain, and caregiver health among Parkinson’s disease (PD) and non-PD caregivers.

	Full Sample	Non-PD Caregivers	PD Caregivers	Between Group Differences
	*n* = 3116	*n* = 3044	*n* = 72	
	%/*M* (*SD*)	%/*M* (*SD*)	%/*M* (*SD*)	
**Caregiver burden**				***
Lowest—1	26%	27%	12%	
2	15%	16%	3%	
3	17%	16%	30%	
4	27%	26%	30%	
Highest—5	15%	15%	25%	
**Caregiving-related strain (range: 3–15)**	7.6 (3.1)	7.6 (3.1)	8.9 (2.9)	***
**Financial strain (range: 1–5)**	2.2 (1.3)	2.2 (1.3)	2.5 (1.3)	
**Emotional stress (range: 1–5)**	3.0 (1.3)	3.0 (1.3)	3.4 (1.1)	**
**Physical strain (range: 1–5)**	2.4 (1.2)	2.4 (1.2)	3.0 (1.2)	***
**Poor/Fair caregiver health**	18%	18%	22%	

Note: *M* = mean, *SD* = standard deviation; ** *p* < 0.01; *** *p* < 0.001.

**Table 3:** Decomposition of total, indirect, and direct effects for the presence of Parkinson’s disease (PD), caregiver burden, caregiving-related strain, and caregiver health.

	Total	Direct	Indirect
PD-->Burden-->Strain-->Poor/Fair Health	0.023	−0.004	0.027 **
PD--> Burden-->Strain	1.254 ***	0.749 *	0.505 ***
Burden-->Strain-->Poor/Fair Health	0.018 **	0.002	0.015 ***

Note: * *p* < 0.05; ** *p* < 0.01; *** *p* < 0.001.

**Table 4:** Decomposition of total, indirect, and direct effects for the presence of Parkinson’s disease (PD), caregiver burden, financial, emotional, and physical strain, and caregiver health.

	Total	Direct	Indirect
PD-->Burden-->Financial Strain-->Poor/Fair Health	0.021	0.004	0.017 *
PD-->Burden-->Financial Strain	0.291	0.149	0.142 ***
Burden-->Financial Strain-->Poor/Fair Health	0.018 **	0.010	0.008 ***
PD-->Burden-->Emotional Stress-->Poor/Fair Health	0.023	0.002	0.021 **
PD-->Burden-->Emotional Stress	0.399 **	0.240	0.159 ***
Burden-->Emotional Stress-->Poor/Fair Health	0.017 **	0.008	0.009 ***
PD-->Burden-->Physical Strain-->Poor/Fair Health	0.023	−0.005	0.028 **
PD-->Burden-->Physical Strain	0.562 ***	0.355 **	0.207 ***
Burden-->Physical Strain-->Poor/Fair Health	0.017 **	0.003	0.014 ***

Note: * *p* < 0.05; ** *p* < 0.01; *** *p* < 0.001.

## Data Availability

The data used in this study were from a combined sample of respondents from the National Alliance for Caregiving’s *Caregiving in the U.S. 2015* and *Caregiving in the U.S. 2020* studies. Data can be obtained at https://www.caregiving.org/research/open-data/, accessed on 17 January 2024.
